# Electrospun Polymer Nanofibers Reinforced by Tannic Acid/Fe^+++^ Complexes †

**DOI:** 10.3390/ma9090757

**Published:** 2016-09-06

**Authors:** Weiqiao Yang, Ana M. M. Sousa, Audrey Thomas-Gahring, Xuetong Fan, Tony Jin, Xihong Li, Peggy M. Tomasula, LinShu Liu

**Affiliations:** 1 Department of Agriculture, Dairy and Functional Foods Research Unit, Eastern Regional Research Center, 600 East Mermaid Lane, Wyndmoor, PA 19038, USA; weiqiao.yang@ars.usda.gov (W.Y.); ana.sousamm@gmail.com (A.M.M.S.); audrey.thomasgahring@ars.usda.gov (A.T.-G.); xuetong.fan@ars.usda.gov (X.F.); tony.jin@ars.usda.gov (T.J.); peggy.tomasula@ars.usda.gov (P.M.T.); 2 Key Laboratory of Food Nutrition and Safety (Ministry of Education), Tianjin University of Science and Technology, Tianjin 300072, China; lixihong606@163.com

**Keywords:** complexation, polyphenol, antioxidant, mechanical reinforcement, electrospinning

## Abstract

We report the successful preparation of reinforced electrospun nanofibers and fibrous mats of polyvinyl alcohol (PVA) via a simple and inexpensive method using stable tannic acid (TA) and ferric ion (Fe^+++^) assemblies formed by solution mixing and pH adjustment. Changes in solution pH change the number of TA galloyl groups attached to the Fe^+++^ from one (pH < 2) to two (3 < pH < 6) to three (pH < 7.4) and affect the interactions between PVA and TA. At pH ~ 5.5, the morphology and fiber diameter size (FDS) examined by SEM are determinant for the mechanical properties of the fibrous mats and depend on the PVA content. At an optimal 8 wt % concentration, PVA becomes fully entangled and forms uniform nanofibers with smaller FDS (*p* < 0.05) and improved mechanical properties when compared to mats of PVA alone and of PVA with TA (*p* < 0.05). Changes in solution pH lead to beads formation, more irregular FDS and poorer mechanical properties (*p* < 0.05). The Fe^+++^ inclusion does not alter the oxidation activity of TA (*p* > 0.05) suggesting the potential of TA-Fe^+++^ assemblies to reinforce polymer nanofibers with high functionality for use in diverse applications including food, biomedical and pharmaceutical.

## 1. Introduction

Polymer fibers and fibrous mats produced by electrospinning technology have attracted considerable interest due to their unique properties not seen in fibers produced by other-fiber forming methods. These properties include: (i) fiber diameter sizes in the nanometer range; (ii) microporous structure; (iii) huge surface area-to-weight ratio which allows a more efficient incorporation, protection and diffusion of functional compounds; and (iv) enhanced mechanical properties [1,2,3,4,5]. During electrospinning, fibers are produced using high voltage through a simple and scalable process that can be applied to a wide diversity of materials. The technical aspects of the technology have been well addressed in a few good reviews [5,6,7].

The search for ways to further improve the mechanical properties of electrospun nanofibers is a current concern among researchers [3,8]. Strategies include polymer blending [9], additives incorporation [10], crosslinking [11] and fiber orientation techniques using collectors specially designed for the effect [12]. More recently, Kim and co-workers reported the mechanical reinforcement of electrospun polycaprolactone nanofibers with Fe^+++^-3,4-dihydroxyphenylalanine (DOPA) complexation by manipulation of solution pH [8]. Improved mechanical properties have been associated with optimal fiber content, fibers with less defects, smaller FDS, and fibers with high orientation [3].

Tannic acid (TA) is a water-soluble, plant polyphenol with natural antioxidant, antimicrobial, and antiviral activities which has interest for practical applications across many different fields [13,14,15,16,17]. TA has been used in electrospinning with polycaprolactone [18], to coat cellulose nanofibers [19] and as a crosslinker for chitosan fibers [20] for potential use in biomedical, pharmaceutical and food applications. As DOPA, TA shows a great binding affinity for metal ions [21] and for many polymers including polyvinyl alcohol (PVA) [22].

**Figure 1:**
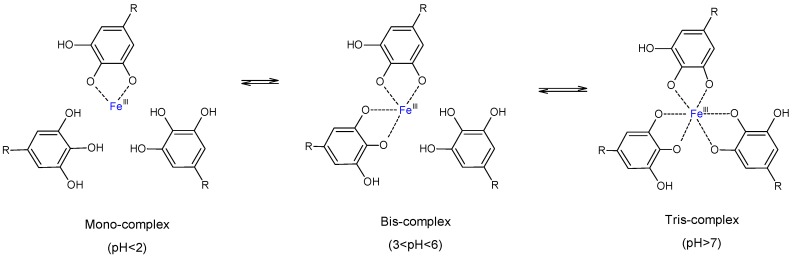
Schematic pH-responsive transition of the TA-Fe^+++^ complex state.

A recent and interesting study from another laboratory [23], reported the formation of self-standing films and capsules of TA-Fe^+++^ complexes using a simple and cost-effective method. The stable TA-Fe^+++^ assemblies were formed by simple mixing of TA and Fe^+++^ aqueous solutions and pH adjustment and were used to coat various substrates yielding self-standing structures after substrate dissolution [23]. By changing solution pH from pH < 2 to 3 < pH < 6 and then to pH > 7, one ferric ion could gradually attach to one, two and three galloyl groups of TA to form respectively, mono-(TA-Fe^+++^ (I)), bi-(TA-Fe^+++^ (II)) and tri-complexes (TA-Fe^+++^ (III)) as shown in Figure 1. These metal-organic ligand coordination assemblies show high versatility and have been tested for many applications including drug delivery [24].

Exploring the concept introduced by Ejima et al. [23], we report a new and inexpensive method to reinforce electrospun polymer nanofibers using TA-Fe^+++^ complexes. PVA was chosen as model polymer due to its biocompatibility, biodegradability, non-toxicity and excellent spinnability [25,26,27]. PVA is also inexpensive and it is used in a broad range of applications including wound healing, tissue engineering and drug delivery [25], while a few studies have focused on food uses [28,29,30]. The idea was to see if the TA-Fe^+++^ assemblies improve the mechanical properties of electrospun polymer nanofibers as seen with DOPA-Fe^+++^ [8]. The morphology, size and mechanical properties of the PVA/TA-Fe^+++^ fibrous mats were examined at different polymer concentrations and solution pH, and related with relevant solution properties for spinning including rheological, electrical conductivity and surface tension. Finally, we examined the effect of the Fe^+++^ in the anti-oxidant properties of TA by comparing mats of PVA/TA-Fe^+++^ with mats of PVA alone and of PVA with TA.

## 2. Materials and Methods

### 2.1. Materials

Polyvinyl alcohol (PVA, weight average molecular weight 85,000–124,000, 84%–89% hydrolyzed), tannic acid (C_76_H_52_O_46_) and iron (III) chloride hexahydrate (FeCl_3_·H_2_O) were purchased from Sigma-Aldrich (St. Louis, MO, USA). Regular refined sunflower oil without synthetic antioxidants was obtained from Cargill, Minnetonka, MN, USA. The initial conjugated diene hydroperoxides (CD) value at 232 nm of the sunflower oil was 4.39%. MilliQ water with a resistivity of 18.2 MΩ. cm was prepared using Barnstead E-pure water system (Dubuque, IA, USA) and used in all experiments.

### 2.2. Methods

#### 2.2.1. Preparation of PVA Stock Solutions and TA-Fe^+++^ Stock Suspension

TA-Fe^+++^ complexes were easily prepared by mixing equal volumes of TA (0.8 mg/mL) and FeCl_3_·H_2_O (0.2 mg/mL) aqueous solutions at room temperature under stirring to prepare a suspension with 0.4 mg/mL of TA and 0.1 mg/mL of Fe^+++^, as described elsewhere [23]. The final solution pH was adjusted to 7.40 using 1.0 N NaOH.

PVA stock solutions with concentrations ranging from 2 to 24 wt % were prepared by dissolving the polymer granules in MilliQ water at 85 °C for one hour under vigorous mechanical agitation using a propeller stirrer (700 rpm, RZR 2041, Heidolph, Schwabach, Germany), and then stored overnight at 4 °C prior to use.

#### 2.2.2. Preparation and Characterization of Spinning Solutions

The spinning solutions were prepared by mixing the PVA stock solutions (2–24 wt %) with the TA-Fe^+++^ suspension at a 1:1 mass ratio. Mixing was promoted at room temperature with the aid of a propeller stirrer at 700 rpm for 30 min. The PVA/TA-Fe^+++^ spinning solutions thus prepared had PVA concentrations between 1 and 12 wt % and fixed TA-Fe^+++^ content of TA (0.2 mg/mL) and Fe^+++^ (0.05 mg/mL).

In other experiments, the pH of a spinning solution with 8 wt % PVA was adjusted to 2.00 or 7.40 using 1.0 N HCl or 1.0 N NaOH, respectively. Spinning solutions of PVA alone and of PVA with TA (0.2 mg/mL) with 8 wt % PVA were used as control experiments.

The spinning solutions were characterized by measuring the rheological properties, surface tension and electrical conductivity. The rheological tests were performed in a Kinexus Rheometer (Malvern Instruments, Worcestershire, UK) with attached cone-plate geometry (40 cm, 2° cone, truncation gap 54 µm). Oscillatory measurements were carried out in the linear viscoelastic region of each solution, determined by strain sweep tests. The viscoelastic properties (elastic modulus, G’, and viscous modulus, G”) as function of angular frequency (ω) were measured through frequency sweep tests over the range, 0.1–500 rad/s. Steady shear measurements were performed in the shear rate range of 0.1–500 s^−1^ to build flow curves from apparent viscosity data of each solution. The surface tension was measured using a Fisher Surface Tension meter Model 20 (Pittsburgh, PA, USA) and the electrical conductivity was measured using a H270G pH/Conductivity/Salinity meter (Hach, Loveland, CO, USA).

All measurements were done in triplicate and carried out at 25 °C.

#### 2.2.3. Electrospinning

Electrospinning was performed using a NaBond NEU-PRO unit (NaBond Technologies Co., Limited, Shenzhen, China). A tubeless spinneret (NaBond Technologies Co., Limited, Shenzhen, China) was attached inside the chamber and connected to an external syringe pump (model TCI-IV, Veryark, Nanning, China). This type of spinneret allows the use of smaller sample volumes since it does not need connecting tubes between the needle and the syringe. A drum collector covered with non-sticky aluminum foil and rotating at 41 rpm was used to collect the fibers. A scheme of the electrospinning set-up used to perform the experiments can be found elsewhere [31]. After several preliminary trials the electrospinning conditions were fixed at: 0.5 mL/h of flow rate (Note: this value set in the syringe pump matches a real flow rate of 0.25 mL/h when using the tubeless spinneret), a 15 cm distance from the needle tip-to-collector and 15 kV of applied voltage. A metallic needle with an outer diameter × length = 0.7 mm × 32 mm and inner diameter = 0.390 mm) attached to a 5 mL plastic syringe (initial sample volume of 3 mL) were used in all experiments.

#### 2.2.4. Fiber Characterization

Fibers and fibrous mats obtained by electrospinning were first characterized in terms of morphology, FDS and size distribution, by scanning electron microscopy (SEM). Next, the mats were mechanically tested to determine their tensile strength (TS) and elongation-at-break (EB), and were also tested for antioxidant activity*.*

For SEM examination, specimens of each fibrous mat were mounted with double-side adhesive tapes on specimen stubs, coated with a thin film of gold, and painted with colloidal silver adhesive. The images were acquired using a Quanta 200 FEG scanning electron microscope (FEI, Hillsboro, OR, USA) in the high-vacuum/secondary electron imaging mode, using accelerating voltages of 10 kV and working distances in the range 10.1–11.4 mm. The FDS of a relevant number of fibers shown in each SEM micrograph were measured using the image analysis software XT Document (FEI Corp, Hillsboro, OR, USA) to build a diameter histogram.

For the mechanical analysis, the fibrous mat were carefully peeled off from the aluminum foil, and cut into rectangular dimensions of width × gauge length = 10 mm × 40 mm. A square paper board frame with dimensions 60 × 60 mm^2^ was used to hold the nanofiber mat specimens and prevent their damage during handling. The thicknesses of each specimen were measured at three different points using a digital micrometer (AMES, 00-0341, Waltham, MA, USA) with a precision of 1 μm and averaged. Specimens thus obtained were examined using a texture analyzer (TA-XT 2, Texture Analyser Technologies Corp., Surrey, UK) with adequate tensile grips attached and using a load cell of 2 Kg. The test method was adapted from the ASTM for tensile properties of thin plastic sheeting (D 882-02) to our type of material. Tensile grips were lined with pressure-sensitive tape to avoid slippage of test specimens. A cross head speed of 19.8 mm/min was used to record the stress vs. strain curves of at least eight specimens for each fibrous mat. The film specimens were stored at room temperature under controlled relative humidity of 53% for three days before the analysis.

An accelerated oxidation method [32] was used in the antioxidant activity experiments. Briefly, 0.030 g of fibrous mats of each sample were placed in an 20 mL airtight vial containing 5.0 g refined sunflower oil, the vial was shielded from light by externally covering it with aluminum foil, then stirred for 30 min at 100 rpm, and after that, incubating it in the dark at 60 °C for 12 days. The lipid hydroperoxide concentration in the oil phase was determined by measuring conjugated diene absorbance. The test was done in triplicate.

#### 2.2.5. Statistical Analysis

Statistical analysis was performed using Statistica 8.0 software (StatSoft, Tulsa, OK, USA). One-way analysis of variance (ANOVA) and Tukey’s multiple comparisons test were performed to determine the significance of differences between the mean values of surface tension, conductivity, viscosity, G”, TS and EB at a *p-*value < 0.05 (for a 95% confidence level). The Shapiro-Wilk W test adapted to large sample sizes by Royston [33] was used for testing normality of FDS (*p* < 0.05). Means of FDS following the normality and homogeneity of variance requirements were compared using Tukey test (*p* < 0.05), whereas Mann-Whitney test (*p* < 0.05) was used for comparing means of FDS with non-normal distributions. Data are represented as mean ± standard deviation.

## 3. Results and Discussion

### 3.1. Formation of TA-Fe^+++^ Complexes

The coordination complexes of TA-Fe^+++^ were rapidly assembled and disassembled by pH manipulation as confirmed by the noticeable changes in solution color from green blue (pH ~ 2) to dark-blue (pH ~ 5), and then to violet-red (pH > 7). These color changes matched three different complexation states reflecting the interactions between TA and Fe^+++^ in water (Figure 1): TA-Fe^+++^ (I) mono-complexes formed at pH < 2; TA-Fe^+++^ (II) bi-complexes formed between pH 3–6, and TA-Fe^+++^ (III) tri-complexes assembled at pH > 7, where each metal ion cross-linked with one, two or three galloyl groups of the polyphenol, respectively. The mechanism has been well described by Ejima et al. [23].

### 3.2. Characterization of the Spinning Solutions

Experimental details of the spinning solutions are given in Table 1.

**Figure 2:**
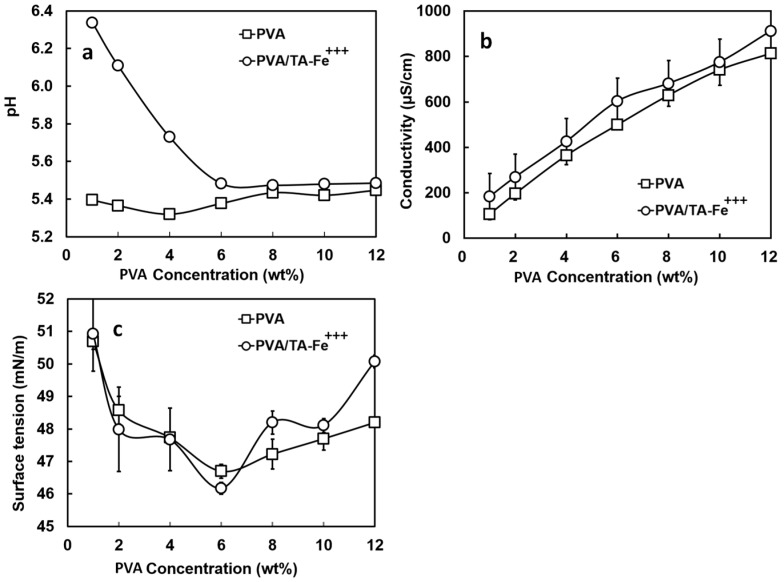
Changes in solution pH (**a**), electric conductivity (**b**) and surface tension (**c**) of PVA (squares) and PVA/TA-Fe^+++^ (circles) solutions as function of PVA concentration.

First, we monitored the solution pH to follow the transition between the different complexation states of TA-Fe^+++^. The pH measurements of the spinning solutions fell between those of the PVA stock solutions (pH ~ 5.5 for PVA concentrations ≥ 6 wt %) and TA-Fe^+++^ (III) stock suspension (pH 7.40) (Figure 2a). At a minimum PVA concentration of 1 wt %, the solution pH of 6.38 was closer to that measured for the neat TA-Fe^+++^ (III) and gradually decreased as the PVA concentration increased, reaching ~5.5 at 6 wt % PVA. Above this concentration, the solution pH remained practically constant. At pH ~ 5.5, the spinning solutions colored dark-blue indicating the formation of TA-Fe^+++^ (II) complexes [23].

Next, we measured the changes in electrical conductivity (b) and surface tension (c) of the spinning solutions of PVA/TA-Fe^+++^ (II) and of PVA alone as the PVA content increased from 1 to 12 wt % (Figure 2). Conductivity and surface tension are important to ensure an adequate electrostatic force that deforms the solution droplet prior to jet formation [34].

Consistent with the results of another group [25], the electrical conductivity gradually increased (*p* < 0.05) from 95 to 800 µS/cm with the increase in PVA concentration. In our view, the contributions for solution conductivity were mainly two: the ferric ions kept at a constant concentration in the spinning solutions and the acetyl groups and other impurities of PVA [25]. While the latter could explain the gradual increase noted in conductivity as the PVA concentration increased, the former agreed well with the higher conductivities measured for the PVA/TA-Fe^+++^ (II) solutions when compared with the solutions of PVA alone (*p* < 0.05). The neat TA-Fe^+++^ suspension had a conductivity of 200 µS/cm, which was close (*p* > 0.05) to the value measured for the spinning solution prepared with lowest PVA concentration of 1 wt %. Above 1 wt % PVA, the measurements were all above 200 µS/cm (*p* < 0.05) as the polymer started to contribute more to solution properties.

As shown in Figure 2c, little variation was seen in surface tension when adding TA-Fe^+++^ (II) to the PVA solutions (46–52 mN/m; *p* > 0.05). As the PVA content in solution increased from 1 to 6 wt %, the surface tension described a descending trend although from 2 to 4 wt % PVA the changes were not significant (*p* > 0.05; ~48 mN/m). Above 6 wt % PVA, we could note an increase in surface tension with PVA content especially from 10 to 12 wt % (*p* < 0.05). At a maximum PVA concentration of 12 wt %, the surface tension was similar to that of the 1 wt % PVA solution (~51 mN/m; *p* > 0.05). A hypothesis was offered to explain this phenomenon [25]; at high PVA concentration, the macromolecules bonded together to form close, internal structures that squeezed water out while in dilute PVA, the macromolecules functioned as a surfactant and were present on the solution surface. In our case, we also need to consider the TA-Fe^+++^ (II) complexes. Supposing that these assemblies functioned like a salt or electrolyte in solution, we could expect the disruption of the hydrogen bonding between PVA and water and a “salting out” effect where more macromolecules would be forced to the solution surface and thus reducing the surface tension. Since the TA-Fe^+++^ content in our spinning solutions was constant, once the “salting out” influence reached its maximum, the surface tension increased again. Opposite to our findings and to the findings of Rošic et al. [25], Rwei and Huang observed a gradual decrease in surface tension as the PVA concentration increased [26].

**Figure 3:**
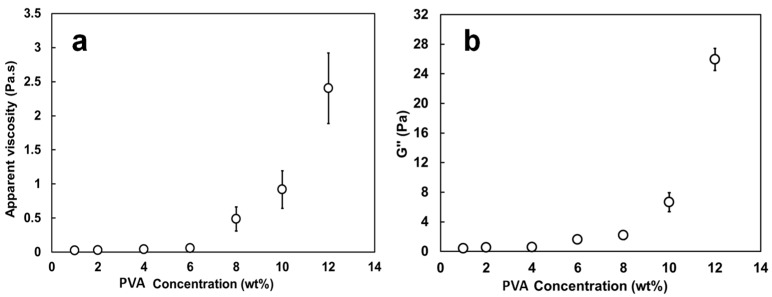
Changes in apparent viscosity measured at 10 s^−1^ (**a**) and viscous modulus G” measured at 1 Hz (**b**) of the PVA/TA-Fe^+++^ solutions as a function of PVA concentration.

Figure 3 shows the values of apparent viscosity (a) and of viscous modulus G” (b) for the PVA/TA-Fe^+++^ (II) solutions when the PVA concentration varied from 1 to 12 wt %.

The apparent viscosities measured at 10 s^−1^ and G” measured at 6.31 rad/s were chosen as indicative of the potential to produce fibers even though the shear encountered in electrospinning is much greater. Solutions of PVA alone showed very similar results (*p* > 0.05) and are not represented. The viscosity (~0.05 Pa·s) and G” (~0.4–1.6 Pa) were low and remained almost unchanged up to 6 wt % PVA. Beyond this point, the addition of more PVA resulted in a sharp increase for both parameters. At a PVA content of 8 wt %, the solutions had a viscosity of 0.49 Pa·s and a G” of 2.18 Pa and as the polymer concentration reached a maximum of 12 wt %, the apparent viscosity and G” reached their highest values of 2.40 Pa·s and ~18 Pa, respectively. The G’ values recorded during oscillatory tests described the same trend as G” but with much lower magnitudes and a high degree of scattering (not shown). The sharp increase noted in viscosity and G” after 6 wt % PVA concentration is consistent with the studies of Rošic and Rwei [25,26] and suggests that 8 wt % is the point at which the PVA chains become fully entangled, i.e., where the inter- and intra-chain hydrogen bonding of the macromolecules are significant enough to cause relevant changes in the rheological properties (Figure 3). Below this concentration, PVA is mostly in an untangled state. Polymer concentration and solution viscosity depend on chain entanglements which will avoid fragmentation of the polymer jet and lead to continuous nanofibers [35] while an adequate solution viscoelasticity with a predominant viscous component (i.e., G” >> G’) will allow jet initiation and elongation [25].

### 3.3. Electrospinning

**Figure 4:**
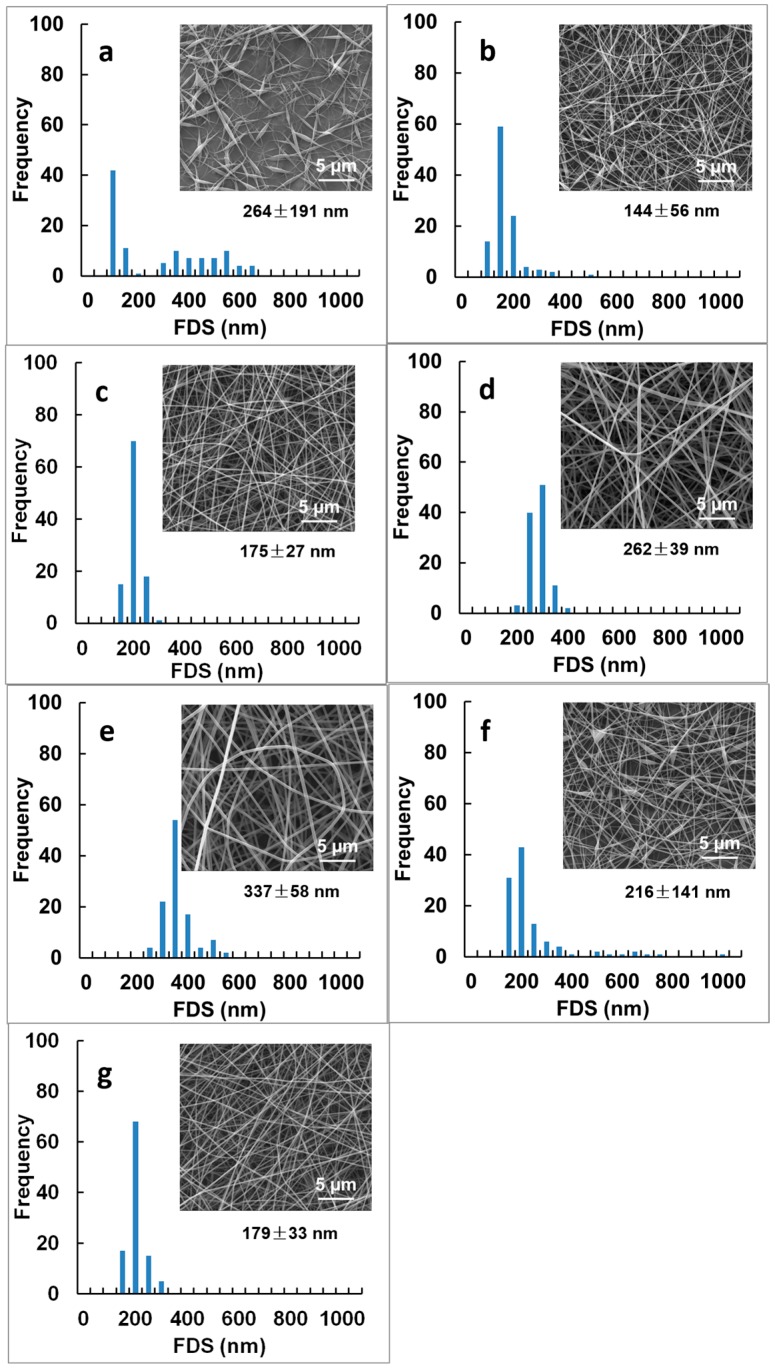
SEM images and fiber diameter size (FDS) of electrospun PVA nanofibers reinforced with TA-Fe^+++^ (II) complexes as function of PVA concentration: (**a**) 4 wt %; (**b**) 6 wt %; (**c**) 8 wt %; (**d**) 10 wt %; and (**e**) 12 wt %; and of (**f**) 8 wt % PVA and (**g**) 8 wt % PVA/TA. Experimental details of the spinning solutions can be found in Table 1. Magnification is 10,000×.

Representative SEM images and histograms of FDS of the fibrous mats prepared from PVA/TA-Fe^+++^ (II) solutions with increasing PVA content are shown in Figure 4.

A strong dependence was seen between polymer concentration and fiber morphology and FDS which is in good agreement with previous electrospinning studies of PVA alone [25]. The minimum PVA concentration that could form fibers was 4 wt % although the fibers were discontinuous and showed many defects (Figure 4a) as confirmed by the broad distribution of FDS between 76 and 455 nm. At lower PVA concentrations, the polymer jet would break up into small drops during spinning and spray-deposit onto the collector as beads (not shown). This suggested that the PVA was mostly in a non-entangled state and agreed well with the low solution viscosity and low G” measured for these samples (Figure 3). Jet stabilities were improved at 6 wt % PVA content (not shown) although the fibers were irregular and the FDS non-normally distributed (*p* < 0.05; Figure 4b). Defect-free fibers with mean FDS of 175 ± 27 nm were formed at 8 wt % PVA (Figure 4c), concentration at which the PVA chains became fully entangled (Figure 3) [36]. The fibers were also more uniform in size as confirmed by the normal distribution of FDS (*p* < 0.05; Figure 4c). A further increase in PVA concentration to 10 wt % and then to 12 wt % increased the FDS to 262 ± 39 nm (*p* < 0.05; Figure 4d) and then to 337 ± 58 nm (*p* < 0.05; Figure 4e) but the fibers became less uniform as indicated by the non-normal distribution of FDS (Figure 4e).

As control experiments, we electrospun solutions of PVA alone and of PVA/TA with 8 wt % PVA, which was the optimal concentration to form uniform and defect-free fibers (Figure 4c). Beaded fibers with irregular FDS ranging between 75 and 357 nm where obtained when spinning the PVA alone (Figure 4f). The inclusion of TA prevented beads formation and improved fiber morphology (Figure 4g) as at low pH, the galloyl groups in TA are more likely to bond with PVA [22]. The obtained FDS were within the same range than those of PVA alone (179 ± 33 nm; *p* > 0.05) and followed a non-normal distribution (*p* < 0.05). Thus, optimal fiber morphology and maximal FDS uniformity was obtained with the PVA/TA-Fe^+++^ (II) mat prepared at 8 wt % polymer concentration (Figure 4c).

**Figure 5:**
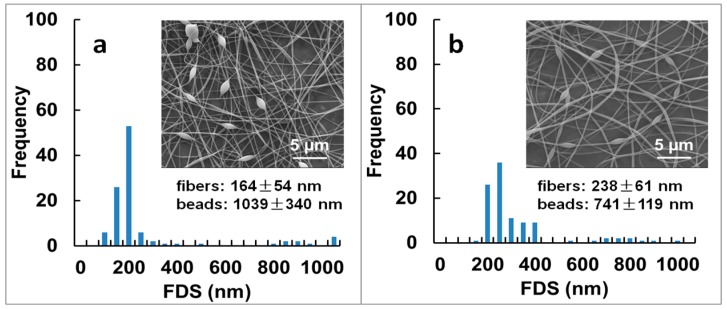
SEM images and fiber diameter size (FDS) of electrospun nanofibers prepared at 8 wt % PVA concentration and reinforced with (**a**) TA-Fe^+++^ (III) and (**b**) TA-Fe^+++^ (I) complexes. Experimental details of the spinning solutions can be found in Table 1. Magnification is 10,000×.

Fibrous mats from spinning solutions with 8 wt % PVA content at different solution pH were also prepared and are shown in Figure 5.

Adjusting the solution pH to 7.40 or to 2.00, to form respectively, tri-(TA-Fe^+++^ (III)) or mono-(TA-Fe^+++^ (I)) complexes, led to the formation of beaded fibers with more irregular distributions of FDS (Figure 5). Both mats showed a region of smaller FDS < 500 nm, excluding beads and other defects between 622 and 1379 nm. Solution parameters [6,37] such as electrical conductivity, which increased almost ten times after pH adjustment to 2.00 (~5.04 mS/cm; *p* < 0.05), and surface tension, that went up to ~51 mN/m, could explain the strong dependence of fiber morphology and FDS with solution pH. Next, we evaluated the effect of TA-Fe^+++^ assemblies in the mechanical properties of the fibrous mats.

### 3.4. Mechanical Properties of the Fibrous Mats

The TS and EB of the electrospun fibrous mats obtained from stress-strain curves recorded in a texture analyzer are shown in Table 2. Solutions with PVA concentrations below 8 wt % did not give self-standing mats, and for this reason, were not tested.

First we examined mats loaded with TA-Fe^+++^ (II) and increasing PVA contents from 8 to 12 wt %. The TS was highest at 8 wt % PVA content (~31.4 MPa against ~17.4 MPa at 10 wt % and ~13.8 MPa at 12 wt %; *p* < 0.05) while the EB was similar to that obtained at 10 wt % PVA (*p* > 0.05; ~24%). Best mechanical properties of electrospun gelatin fibrous mats were also observed at an intermediate polymer concentration [38]. It is believed that fiber content, fiber morphology and FDS affect the mechanical properties of electrospun fibers and fibrous mats [3], and up to a FDS threshold, smaller electrospun fibers show significant mechanical reinforcement in relation to their larger-size equivalents [3]. This is consistent with our findings (Figure 4c–e). Fibers with 8 wt % PVA were thinner and more uniform (175 ± 27 nm; Figure 4c) than those prepared with higher PVA contents (e.g., 262 ± 39 nm obtained at 10 wt % PVA; Figure 4d). Consistent with other electrospinning studies [39,40], our best mat showed simultaneously high TS and EB. This could relate with the mechanism of load transfer during mechanical tests [12]; under tension, the non-aligned PVA fibers will rearrange due to the load exerted on the fiber network (and not on individual fibers as in the case of aligned fibrous mats) which will cause the fibrous mats to elongate more or less depending on the number of fiber ends the tensile grips will grab.

The TA-Fe^+++^ (II) incorporation increased the TS of the 8 wt % PVA mat by about 70% and doubled the EB to ~24% (against TS ~ 18.6 MPa and EB ~ 11.5% for the mat of PVA alone) which agreed well with the optimal fiber morphology and FDS seen in Figure 4c. The addition of TA did not change much the TS and EB of the PVA mat (*p* > 0.05; Table 2) confirming that the mechanical reinforcement could only be obtained upon Fe^+++^ incorporation (*p* < 0.05; TS ~ 31.4 MPa).

The effect of solution pH in the mechanical properties of the fibrous mats was also examined at 8 wt % PVA content by adjusting the pH of the PVA/TA-Fe^+++^ (II) solution to 7.40 or to 2.00 (Table 1 and Table 2). The PVA/TA-Fe^+++^ (III) mat prepared at pH 7.40 (Figure 5a) showed lower TS (~26.4 MPa) and EB (~16%) than the PVA/TA-Fe^+++^ (II) mat prepared at pH 5.50 (*p* < 0.05; ~31.4 MPa and ~24%, respectively). This agreed well with the changes from beaded-free to beaded fibers seen when increasing the solution pH (Figure 4c vs. Figure 5a). Although TA-Fe^+++^ assemblies are expected to be more stable at pH 7.40 than at pH 5.50 [23], we need to consider the H-bond interactions between TA and PVA. At basic pH, TA has the ability to remain protonated to interact with PVA yet optimal interactions between PVA and TA are seen at pH ~ 4.0 [22]. The PVA/TA-Fe^+++^ (I) prepared at pH 2.00, also composed of beaded fibers (Figure 5b), showed much lower TS (~6 MPa) and EB (~4%) (*p* < 0.05). This was consistent with Fe^+++^ reacting with one TA galloyl group (in opposition to multi galloyl groups at higher solution pH) and with the formation of uncontrollable TA-PVA assemblies when decreasing significantly the pH [28].

To conclude this section, a comment is need on the amount of TA-Fe^+++^ used in the experiments. During our trials, we observed that small amounts of TA (0.2 mg/mL) and Fe^+++^ (0.05 mg/mL) produced significant improvements in the mechanical properties of the mats. Similar effect was seen with other polymers tested in our laboratory (unpublished results). Probable reasons for these results are the increased surface efficiency of electrospun nanofibers, the high binding affinity of TA and high stability of TA-Fe^+++^ assemblies at high solution pH. However, more research is needed to understand these mechanisms. When the TA-Fe^+++^ concentration was increased up to five times, the properties of the PVA/TA-Fe^+++^ solutions changed significantly due to Fe^+++^ excess, which resulted in poorer spinnability (not shown).

### 3.5. Antioxidant Activity Assay in Sunflower Oil

**Figure 6:**
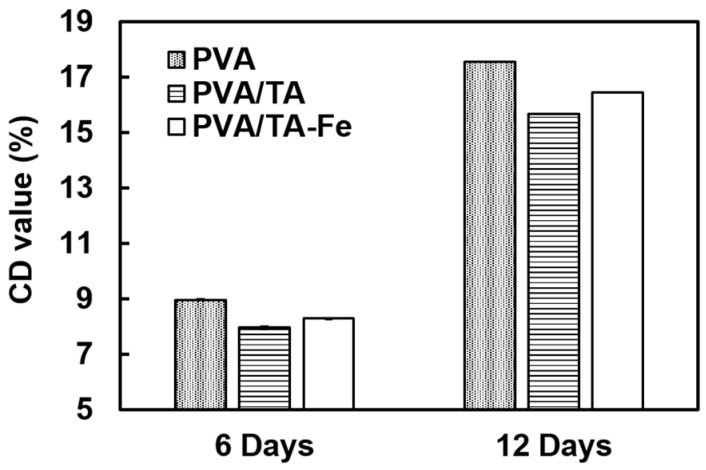
Antioxidant activity of fiber mats prepared with 8 wt % PVA concentration in sunflower oil.

The antioxidant activity of the optimum PVA/TA-Fe^+++^ (II) fibrous mat was investigated by measuring the amount of conjugated diene, the primary product of lipid oxidation, in an accelerated model using sunflower oil with and without the mat. As control experiments, we tested mats of PVA with TA and of PVA alone to see how the Fe^+++^ affected the antioxidant activity of TA. As shown in Figure 6, the PVA/TA-Fe^+++^ (II) mat was able to decrease the formation of conjugate dienes, especially for long term storage. Most importantly, the Fe^+++^ incorporation did not affect much the antioxidant activity of TA (*p* > 0.05). Obviously, the antioxidant activity of the mats could be increased by increasing the TA concentration but excess of Fe^+++^ should be avoided since it affects significantly the solution parameters and, hence, fiber formation.

## 4. Conclusions

Stable TA-Fe^+++^ assemblies were used to mechanically reinforce polymer nanofiber mats via a simple and cost-effective electrospinning method. At pH ~ 5.5 and for an optimal PVA concentration of 8 wt %, PVA becomes fully entangled, forming uniform and defect-free nanofibers with improved mechanical properties when compared to mats of PVA alone and of PVA with TA. Changes in solution pH result in beads formation, more irregular FDS and poorer mechanical properties of the fibrous mats. The Fe^+++^ does not affect the oxidation activity of TA which suggests the potential of TA-Fe^+++^ assemblies to produce reinforced electrospun polymer nanofibers with high functionality.

## Figures and Tables

**Table 1:** Experimental details of the spinning solutions.

Sample ID.	(PVA) (wt %)	(TA) (mg/mL)	(Fe^+++^) (mg/mL)	Final pH	TA-Fe^+++^ Complexes Preferentially Formed in Solution
4 wt % PVA/TA-Fe^+++^ (II)	4	0.2	0.05	5.7	bi-complexes
6 wt % PVA/TA-Fe^+++^ (II)	6	0.2	0.05	5.5	bi-complexes
8 wt % PVA/TA-Fe^+++^ (II)	8	0.2	0.05	5.5	bi-complexes
10 wt % PVA/TA-Fe^+++^ (II)	10	0.2	0.05	5.5	bi-complexes
12 wt % PVA/TA-Fe^+++^ (II)	12	0.2	0.05	5.5	bi-complexes
8 wt % PVA	8	0	0	5.5	n.a.
8 wt % PVA/TA	8	0.2	0	5.5	n.a.
8 wt % PVA/TA-Fe^+++^ (III) ^1^	8	0.2	0.05	7.4	tri-complexes
8 wt % PVA/TA-Fe^+++^ (I) ^2^	8	0.2	0.05	2.0	mono-complexes

pH adjustment to 7.40 ^1^ and 2.00 ^2^ after mixing the PVA stock solution with the TA-Fe^+++^ suspension at 1:1 mass ratio. n.a.—not applied.

**Table 2:** Mechanical properties (tensile strength, TS (MPa); elongation-at-break, EB (%)) of the nanofiber mats prepared from the spinning solutions listed in Table 1. Mats were prepared with variable PVA concentration and fixed amount of TA-Fe^+++^ complexes. Nanofiber mats of 8 wt % PVA and 8 wt % PVA/TA were used as control experiments.

Sample ID.	TS (MPa)	EB (%)	FDS (nm)	Fiber Morphology
4 wt % PVA/TA-Fe^+++^ (II)	n.d.	n.d.	264 ± 191 ^a,e,f^	discontinuous fibers w/defects
6 wt % PVA/TA-Fe^+++^ (II)	n.d.	n.d.	144 ± 56 ^a^	fibers with defects
8 wt % PVA/TA-Fe^+++^ (II)	31.4 ± 2.5 ^a^	23.9 ± 5.1 ^a^	175 ± 27 ^b^	fibers
10 wt % PVA/TA-Fe^+++^ (II)	17.4 ± 3.8 ^b,c^	24.2 ± 5.4 ^a,b^	262 ± 39 ^c^	fibers
12 wt % PVA/TA-Fe^+++^ (II)	13.8 ± 2.8 ^b^	41.5 ± 2.3 ^c^	337 ± 58 ^d^	fibers
8 wt % PVA	18.6 ± 4.2 ^d^	11.5 ± 4.1 ^d,e^	216 ± 141 ^e^	beaded fibers
8 wt % PVA/TA	22.5 ± 3.3 ^c,d^	17.0 ± 6.9 ^b,d^	179 ± 33 ^e^	fibers
8 wt % PVA/TA-Fe^+++^ (III)	26.4 ± 3.1 ^d^	15.9 ± 3.6 ^b,d^	246 ± 280 ^e^	beaded fibers
8 wt % PVA/TA-Fe^+++^ (I)	5.7 ± 1.4 ^e^	4.0 ± 1.2 ^e^	291 ± 171 ^f^	beaded fibers

Means within a column with different letters (a–f) are statistically different (*p* < 0.05). n.d.—not determined.
